# Increasing stratification as observed by satellite sea surface salinity measurements

**DOI:** 10.1038/s41598-022-10265-1

**Published:** 2022-04-15

**Authors:** Estrella Olmedo, Antonio Turiel, Verónica González-Gambau, Cristina González-Haro, Aina García-Espriu, Carolina Gabarró, Marcos Portabella, Ignasi Corbella, Manuel Martín-Neira, Manuel Arias, Rafael Catany, Roberto Sabia, Roger Oliva, Klaus Scipal

**Affiliations:** 1 Barcelona Expert Center (BEC), Institute of Marine Sciences (ICM) and Consejo Superior de Investigaciones Científicas (CSIC), 08003 Barcelona, Spain; 2 Barcelona Expert Center (BEC), Universitat Politècnica de Catalunya (UPC), 08034 Barcelona, Spain; 3 European Space Agency, ESTEC, Noordwijk, 2201 AZ The Netherlands; 4 Argans UK Ltd., Plymouth, PL6 8BX UK; 5 Telespazio-UK, for ESA-ESRIN, 00044 Frascati, Italy; 6 Zenithal Blue Technologies, 08023 Barcelona, Spain

**Keywords:** Climate sciences, Ocean sciences, Energy science and technology, Physics

## Abstract

Changes in the Earth’s water cycle can be estimated by analyzing sea surface salinity. This variable reflects the balance between precipitation and evaporation over the ocean, since the upper layers of the ocean are the most sensitive to atmosphere–ocean interactions. In situ measurements lack spatial and temporal synopticity and are typically acquired at few meters below the surface. Satellite measurements, on the contrary, are synoptic, repetitive and acquired at the surface. Here we show that the satellite-derived sea surface salinity measurements evidence an intensification of the water cycle (the freshest waters become fresher and vice-versa) which is not observed at the in-situ near-surface salinity measurements. The largest positive differences between surface and near-surface salinity trends are located over regions characterized by a decrease in the mixed layer depth and the sea surface wind speed, and an increase in sea surface temperature, which is consistent with an increased stratification of the water column due to global warming. These results highlight the crucial importance of using satellites to unveil critical changes on ocean–atmosphere fluxes.

## Introduction

About $85\%$ of the Evaporation (E) and $77\%$ of the Precipitation (P) occurs over the ocean^1,2^. Both processes produce changes in sea surface salinity (SSS) leading to positive (evaporation) and negative (precipitation) anomalies. In a global warming scenario, the global water cycle is expected to be intensified^3–9^ and is a cause of great concern, because of its profound socioeconomic impacts throughout the globe. Monitoring the SSS to assess the intensification of the water cycle is proposed in Yu et al.^10^ and references therein, as an alternative to directly measure E and P since these components can only be estimated with limited accuracy. However, there is still some controversy as to whether the salinity is changing at the same rate as the water cycle does^11–15^, as the impact of the changes in E–P fluxes, meltwater runoff, and ocean warming on the salinity is not completely understood^16–18^. Moreover, the number of available salinity measurements has been historically scarce and limited to some specific ocean regions^19^. Since 2000, the global array of temperature and salinity floats provided by the Argo system^20^, besides other permanent or routine observation systems, have contributed to further the knowledge on ocean salinity related processes. More recently, since 2010, SSS measurements are also available from space^21–23^, increasing the monitoring capability of this Essential Climate Variable. One of the main differences between satellite and in situ salinity measurements is that the latter are typically acquired at a few meters depth (5–10 m), thus monitoring the near surface salinity (NSS), while the former are providing measurements at the top cm layer of the ocean, thus monitoring the actual SSS.

Differences between the SSS and NSS are due to the vertical stratification of the ocean upper layers. Whereas vertical stratification in temperature has been extensively studied over the past several decades^24,25^, upper-ocean salinity stratification studies were only initiated in recent years, mostly motivated by the analysis of satellite-derived SSS data^26,27^. The ocean salinity stratification results from a complex combination of various mechanisms such as precipitation, oceanic advection and mixing conditions, as well as fresh water input from rivers runoff, melting of sea ice and removal of freshwater through evaporation. Although negative salinity anomalies have been shown and studied in cases of rainfall^28–36^, river runoff^37–39^ and sea-ice melting^40–42^, there is a limited number of studies of positive salinity anomalies due to evaporation^43–45^. Under absence of rainfall, continental discharge or sea ice melting, the upper ocean layer is characterized by a nearly uniform density, active vertical mixing and a high rate of turbulent dissipation^46,47^. In that case, vertical salinity gradients in the upper 10 m are expected to be small^29,35^.

Here, we show that the dynamics captured by satellite SSS measurements actually differ from the dynamics shown by in situ NSS measurements. On one hand, satellite SSS data present a clear intensification of the water cycle which is not so clearly present in the NSS data. On the other hand, we find significant differences between SSS and NSS trends, which suggest that global warming is inducing an increasing stratification over wide open ocean areas.

## Results

### Satellite versus in situ salinity measurements

**Figure 1:**
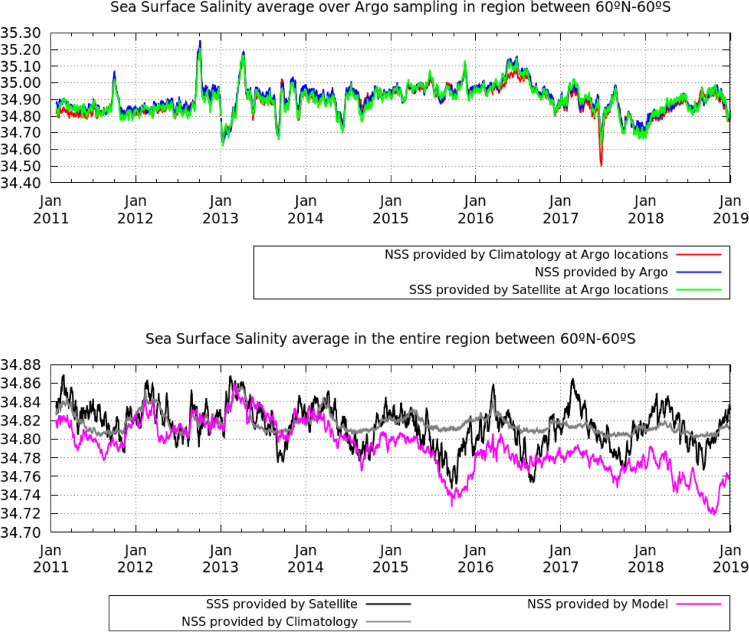
Temporal evolution of the averaged salinity in between $60^\circ$ S and $60^\circ$ N. Top plot: The mean salinity measured by Argo floats (blue), the annual climatology averaged at the Argo locations (red), the satellite salinity averaged at the Argo locations (green). Bottom plot: The satellite salinity (black), salinity provided by model (pink) and annual climatology (grey) averaged over the entire region. In the bottom plot the average domain is common and is given by the satellite coverage. The variations in the annual climatology (grey line, bottom plot) correspond to the variations in the satellite coverage that mainly corresponds with the changes in the sea-ice mask.

Since the year 2000, the observation system of free drifting Argo profiling floats has been increasing, reaching close to 4000 buoys that are nowadays available. The Argo system not only provides the capability for monitoring the salinity dynamics, but also represents the main source of data used for validating satellite measurements and a very valuable input for improving ocean models. However, the distribution of these measurements is not homogeneous over the global ocean. Particularly, coastal and polar regions are under-sampled. Moreover, far from the coast and the poles, the ocean currents drive the locations of the floats, and, thus, the locations of the Argo acquisitions. Over wide oceanic areas, as that comprised between $60^\circ$ S and $60^\circ$ N, the averaged salinity at the Argo locations in a 9-day window evolves with time and it is very different from the temporal evolution of the mean salinity in the entire region, as observed in Fig. 1. In this region, the temporal evolution of the average of the salinity provided by the Argo floats at their sampling locations and collocated satellite data is very similar (see top plot of Fig. 1), which indicates consistency between in situ and satellite measurements. Curiously, the temporal evolution of the averaged salinity over the Argo locations provided by the annual climatology also provides a very similar behaviour (red line in the top plot of Fig. 1), which suggests that this average is strongly conditioned by the the variations of the in situ sampling rather than by the variability of the measured salinity.

**Figure 2:**
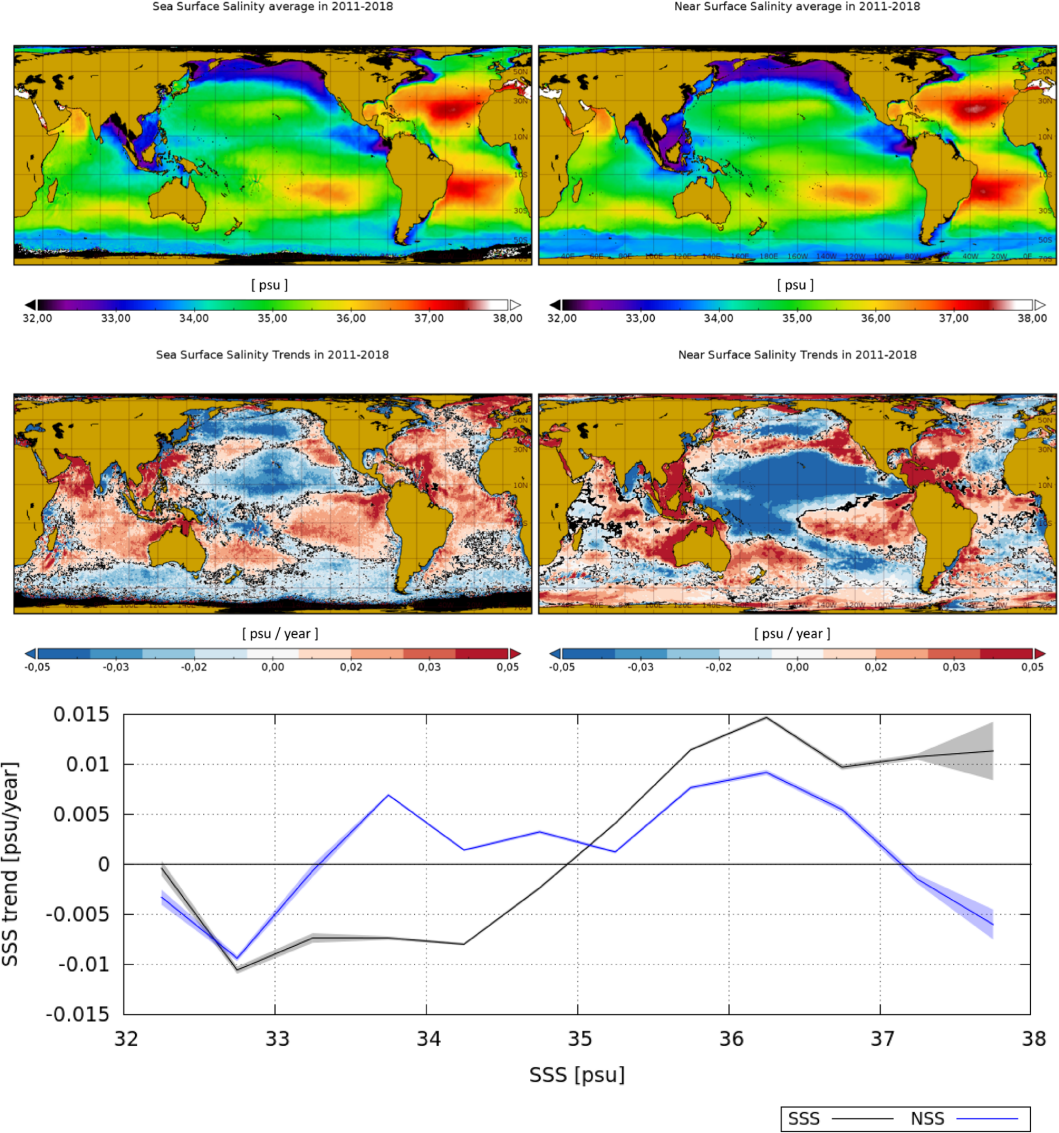
Top row: salinity average in 2011–2018 as observed by the satellite (SSS) (left) and by the model (NSS) (right). Middle row: satellite SSS trends (left) and model NSS trends (right) in 2011–2018. Locations with trends being different from zero with a $95\%$ level of confidence are represented in black. Bottom plot: mean SSS (black) and NSS (blue) trend as a function of averaged SSS and NSS (respectively) in the same period. The shadowed area represents the confidence interval of the $95\%$. Maps are plotted with Panoply v 4.12.0 (https://www.giss.nasa.gov/tools/panoply/).

**Figure 3:**
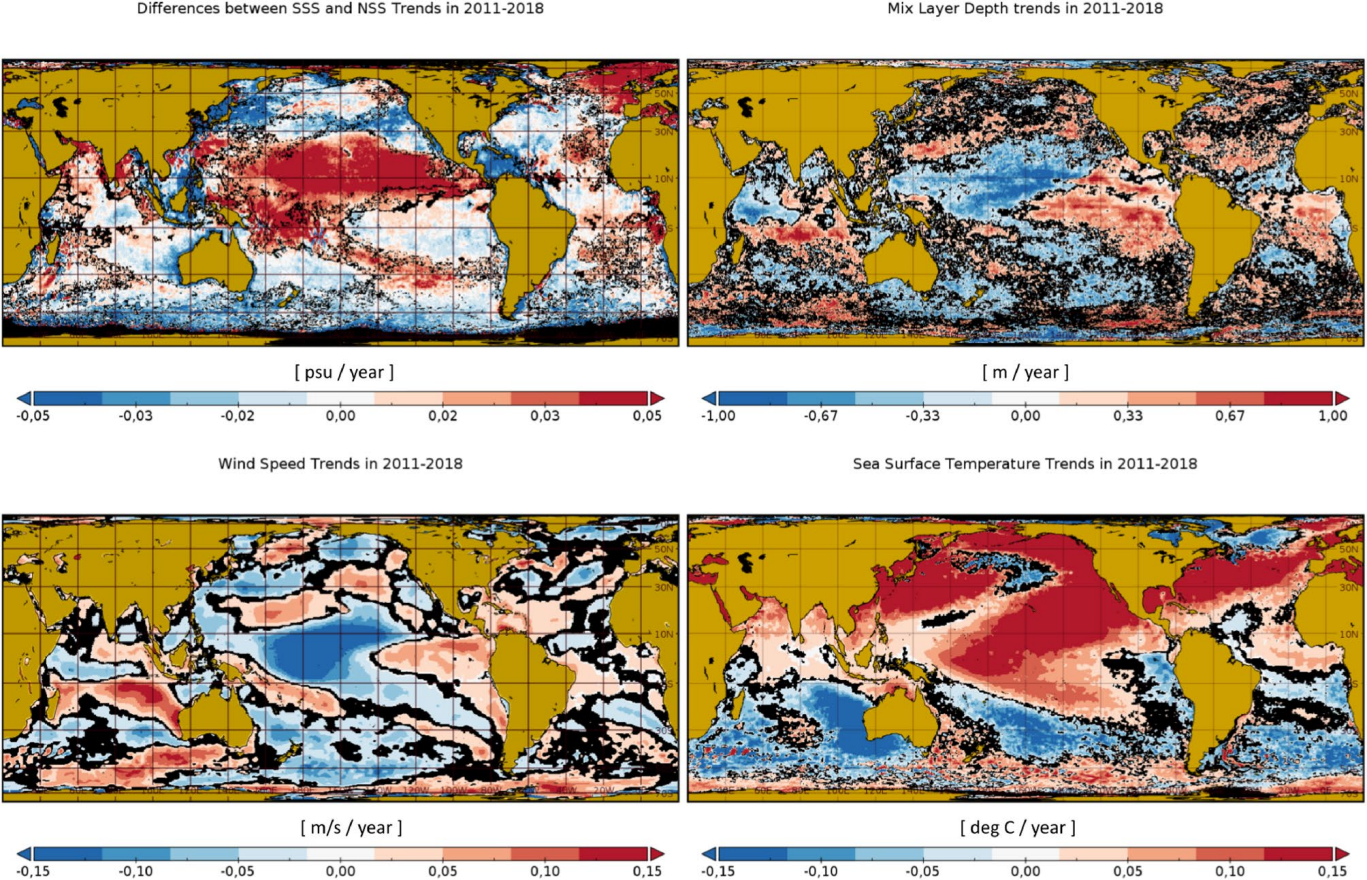
Top left panel: Differences between the satellite SSS trends and the model NSS trends in 2011–2018. Top right panel: mixed layer depth trends in 2011–2018. Bottom row: wind speed trends (left) and sea surface temperature trends in the same period (right). Locations with trends being different from zero with a $95\%$ level of confidence are represented in black. Maps are plotted with Panoply v 4.12.0 (https://www.giss.nasa.gov/tools/panoply/).

**Figure 4:**
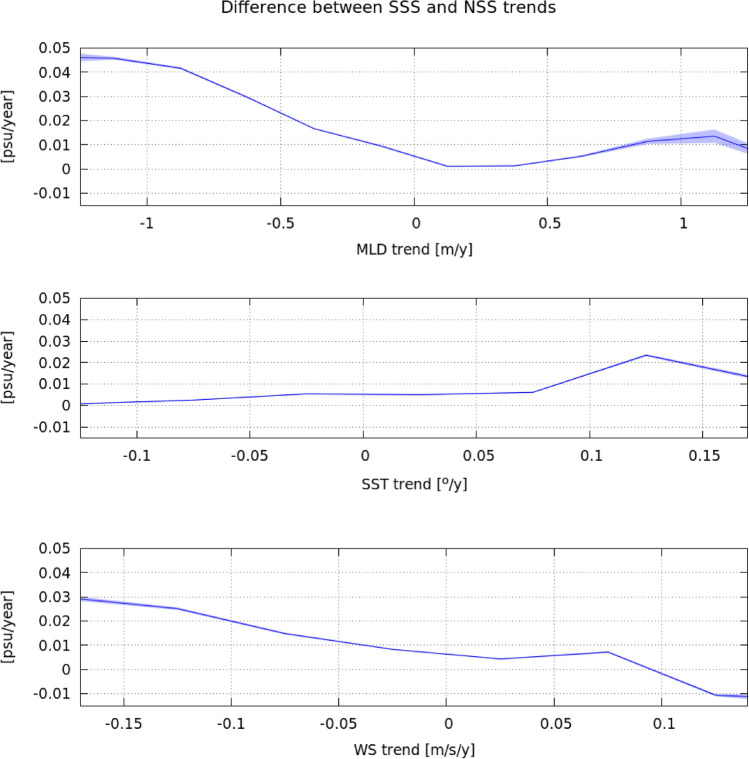
Mean difference between SSS and NSS trends as function of the following trends: mixed layer depth (top panel), sea surface temperature (middle panel), and wind speed (bottom panel). The region considered comprises tropics and middle latitudes (i.e., between $40^\circ$ N and $40^\circ$ S) to exclude from this analysis ocean regions that may be affected by sea-ice dynamics.

The average of the salinity provided by the satellite (black curve in the bottom plot of Fig. 1) and the model (that assimilates Argo, grey curve in the bottom plot of Fig. 1) over the entire region presents a seasonal modulation, which is absent in the salinity average over the Argo sampling locations (see top plot in Fig. 1). This modulation is caused by sea-ice melting and river runoff, which have a seasonal behaviour that is typically under-sampled by the Argo floats and other open-ocean processes that Argo floats sample poorly due to the short residence times of any drifter in some open-ocean regions. Besides, we observe significant differences between the average of the salinity provided by the satellite and the model over the entire region. Part of the differences may come from these under-sampled regions where the model performance may be degraded due to the lack of in situ observations assimilated. Another cause of this difference may come from the fact that the model is providing the NSS and the satellite is providing the SSS, as we will discuss in the following sections.

### Salinity trends in the global ocean

During the 8-year period of study (2011–2018), the maps of averaged salinity for Argo, satellite and model present similar spatial patterns (see first row Fig. 2, averaged Argo salinity is shown in Supplementary Fig. 1). The differences in salinity trends provided by Argo floats and model (see data sets and methods description) are small and mainly due to the differences in the spatial resolutions of the maps (see Supplementary Fig. 1 and Fig. 2, respectively). This is mainly because this model is assimilating Argo salinity data. However, there are significant differences in the trends observed by satellite (SSS) as compared to those observed by in situ and model (NSS). For the rest of the study we use model data because it provides better sampling of the salinity than the Argo data. The averaged satellite salinity trend per isoline or salinity bin (bottom plot of Fig. 2, black line) reveals that fresher regions are getting fresher and saltier ocean regions are getting saltier. Positive trend values, ranging between 0.015 and 0.01 psu/year, are mainly located over regions with salinity values greater than 34.7 psu, while the negative trends, around 0.01 psu/year, are located over regions with salinity values smaller than 34.7 psu. This intensification of the fresher and saltier regions is not so clearly present in the NSS (blue curve). By comparing the geographical distribution of the SSS and NSS trends (second row of Fig. 2, left and right panels respectively), we observe that there are several regions where the discrepancy is significant, among them: (i) the Southern Ocean (salinity values lower than 34.7 psu), where SSS mostly presents negative trend values while NSS presents positive values; (ii) the Atlantic Ocean (salinity values larger than 34.7 psu), where the situation is the opposite, i.e., SSS mostly presents positive trend values while NSS presents both positive and negative values (i.e., no specific predominance of positive trend values).

### Stratification observations in the global ocean

Differences between SSS and NSS trends (top left panel in Fig. 3) reveal a wide ocean region in the Pacific Ocean (comprised between $30^\circ$ S and $10^\circ$ S) where the freshening trend of SSS is significantly weaker than that of NSS. Over the same region, the Mixed Layer Depth (MLD) and the ocean Wind Speed (WS) present negative trends, while the SST presents a positive trend (see Fig. 3). In the region comprised between $40^\circ$ S and $40^\circ$ N, which includes the previous mentioned region in the Pacific, we observe that the largest positive differences between SSS and NSS trends occur when the MLD trend presents the largest negative values, i.e. around − 1 (m/y) (top plot in Fig. 4). In those regions where the MLD is constant or is becoming deeper (null or positive trend), the differences between SSS and NSS trends become small. Negative differences between SSS and NSS trends are compensated by much more frequent positive ones, leading to positive values when they are averaged over any region defined by a fixed value of MLD trend; therefore, the panel on top of Fig. 4 exhibits only positive values of the SSS-NSS average trend. On the other hand, the regions with the largest positive differences between SSS and NSS trends are characterized by a large SST trend [between 0.1 ($^\circ$C/y) and 0.15 ($^\circ$C/y)], while those with SST trends lower than 0.1 ($^\circ$C/y) typically present small differences between SSS and NSS trends. Also note that the largest positive differences between SSS and NSS trends mainly correspond to the largest negative WS trends [between − 0.2 and − 0.15 (m/s/y)]. In regions where the WS trend is increasing differences between SSS and NSS trends become very small or slightly negative.

## Discussion

Satellite measurements are providing a unique source of information of the ocean mesoscale processes in the upper-layer, which cannot be provided by any other means (either models or in situ). They provide routine, global maps of salinity, reaching coastal and polar regions which significantly contribute to the understanding of the sea surface salinity dynamics. Besides satellites measure SSS which is actually different from the NSS measured by the in situ or given by models. Satellite measurements are, therefore, complementary to those provided by in situ.

The water cycle is expected to intensify in a global warming context according to the Clausius–Clapeyron (CC) relation, which states that the saturation of the water vapor pressure increases at a rate of $7\%$ per degree Celsius of warming^4^. The same rate of increase is also expected in Evaporation minus Precipitation over the ocean (as stated Eq. 3 in Yu et al.^10^). This leads to the paradigm of “Dry gets Drier and Wet gets Wetter” (DDWW) under conditions of climate warming. Our results show that the SSS positive trend dominates in regions with SSS larger than 34.7 psu, and the global average is a positive trend, while the opposite is true for regions with SSS lower than 34.7 psu, which is consistent with the DDWW paradigm. In contrast, the NSS doesn’t show this amplification. This reinforces the idea of using SSS (rather than NSS) as a proxy for E–P.

In tropical and mid-latitude regions we observe significant differences between SSS and NSS trends that are probably originated by a net stratification effect induced by surface warming. The persistent increase in temperature under low wind conditions is forming a warm layer in the top few meters of the ocean where the temperature increases towards the surface. Since these conditions persist over time, the evaporation from the ocean surface is favoured. This leads to an increase in SSS with respect to NSS.

## Methods

### Data sets

#### Satellite salinity

We use the Soil Moisture and Ocean Salinity (SMOS) SSS maps generated at the Barcelona Expert Center (BEC, http://bec.icm.csic.es). The European SMOS mission has been continuously providing SSS measurements since 2010^21^. We use the BEC SMOS SSS global product v2^48^, which consists of the 2011–2018 time series of 9-day level 3 salinity maps generated daily at a $0.25^\circ \times 0.25^\circ$ grid. The salinity retrieval procedure does not use in situ salinity measurements for calibration^49^. The main calibration assumption consists of assuming that the global average of SSS does not change with time (the only expected variations are due to changes in the sea-ice extension). Therefore, at each map, the global average of the SMOS salinity is set to be the global average of annual salinity climatology (see^48^ for the details of the methods used in the generation of the SMOS SSS product) . This product is freely available at: http://bec.icm.csic.es/bec-ftp-service/.

#### Sea surface salinity climatology

We use as a salinity reference the annual climatological salinity value provided by the World Ocean Atlas 2013 (WOA2013) at $0.25^\circ \times 0.25^\circ$^50^. We use the average decadal product, which is accessible at the National Oceanographic Data Center (https://www.nodc.noaa.gov/cgi-bin/OC5/woa13/woa13.pl).

#### In situ salinity

We use in situ salinity data obtained by Argo profilers. Argo data are collected and made freely available by the International Argo Program and the national programs that contribute to it (http://www.argo.ucsd.edu, http://argo.jcommops.org). The Argo Program is part of the Global Ocean Observing System. To compare in situ and satellite measurements, we use the same approach as the one described in Olmedo et al.^48^. We temporally and spatially collocate SMOS and Argo data as follows: every map is compared with the Argo salinity acquisition available during the the 9 days of that map. We apply the following quality control over the values of Argo measurements: (i) The cut-off depth for Argo profiles is taken between 5 and 10 m; (ii) Profiles included in the greylist (i.e., floats which may have problems with one or more sensors) are discarded; (iii) We use WOA2013 as a quality indicator: Argo float profiles with anomalies larger than 10 $^\circ$C in temperature or 5 psu in salinity when compared to WOA2013 are discarded; (iv) Only profiles having temperature acquisitions close to surface between − 2.5 and 40 $^\circ$C and salinity between 2 and 41 psu are used.

#### Wind data

We use eight years (2011–2018) of the wind module provided by the IFREMER CERSAT Global Blended Mean Wind Fields. A complete description of the product can be found in https://resources.marine.copernicus.eu/product-detail/WIND_GLO_WIND_L4_REP_OBSERVATIONS_012_006/INFORMATION. Here we include part of this description. ” The estimation of the 6-hourly blended wind products makes use of all of the remotely sensed surface winds derived from scatterometers and radiometers available at this time and use as observation inputs for the objective method dealing with the calculation of 6-hourly wind fields over the global oceans. L4 winds are calculated from L2b products in combination with ERA interim wind analyses from January 1992 onwards. The analysis is performed for each synoptic time (00h:00; 06h:00; 12h:00; 18h:00 UTC) and with a spatial resolution of $0.25^\circ$ over the global ocean.” This product is freely available at: https://resources.marine.copernicus.eu/?option=com_csw&view=details&product_id=WIND_GLO_WIND_L4_REP_OBSERVATIONS_012_006.

#### Ocean model

We use the GLORYS12V1 product. A complete description of this product can be found in https://resources.marine.copernicus.eu/product-detail/GLOBAL_REANALYSIS_PHY_001_030/INFORMATION. Here we include part of this description. ”The Copernicus Marine Service (CMEMS) global ocean eddy-resolving ($1/12^\circ$ horizontal resolution and 50 vertical levels) reanalysis covering the altimetry era 1993–2018. It is based largely on the current real-time global forecasting CMEMS system. The model component is the NEMO platform driven at the surface by ECMWF ERA-Interim reanalysis. Observations are assimilated by means of a reduced-order Kalman filter. Along track altimeter data (Sea Level Anomaly), satellite Sea Surface Temperature, Sea Ice Concentration and in situ temperature and salinity vertical profiles are jointly assimilated. Moreover, a 3D-VAR scheme provides a correction for the slowly-evolving large-scale biases in temperature and salinity. This product includes daily files of temperature, salinity, currents, sea level, mixed layer depth and ice parameters from the top to the bottom. The global ocean output files are displayed on a standard regular grid at $1/12^\circ$ (approximatively 8 km) and on 50 standard levels. In this study we use the salinity provided at 0.5 m depth. The model does not present any change in the salinity trends in the first 4 m depth.” This product is freely available at: https://resources.marine.copernicus.eu/?option=com_csw&view=details&product_id=GLOBAL_REANALYSIS_PHY_001_030.

### Methods

#### Computation of salinity averages

The salinity averages shown in Fig. 1 are computed as follows:

- Salinity average provided by Argo floats (blue line): the average of the salinity provided by the available Argo in the 9-day period used in the generation of the satellite salinity map. Argo measurements are filtered as previously described.
- Climatology (red) and satellite (green) average at the Argo locations: the average of the salinity value in those locations where Argo acquisitions are available.
- Average of the climatology (grey), model (pink) and satellite (black) over the entire region: it is computed as a weighted average of the salinity in those cells where satellite data is available. The weighted function accounts for the area in km$^2$ of each cell:
  1 $$\begin{aligned} s=\frac{1}{\sum _{i\in D} w_i}\sum _{i\in D} w_i s_i, \end{aligned}$$
  where *D* corresponds to the set of cells where satellite data is available, $s_i$ the salinity value of cell *i*, and $w_i$ the extension in km$^2$ of the cell *i*.

#### Computation of trends

We compute the trends as the linear regression coefficient of the temporal series of the salinity value at each cell of the map, by using the following equation:

2 $$\begin{aligned} a=\frac{N<x t>-<x> <t>}{N<t^2>-<t>^2}, \end{aligned}$$

where *N* is the number of elements *x* in the temporal series, *t* the time value of each *x* element in the series and $<\cdot>$ the sum of all the elements of the series. We apply a T-student significance test for the computed trends, such that only the trends with a significance larger than 0.95 are considered in this study. Therefore, we only consider valid a trend value, when $N>100$ and:

3 $$\begin{aligned} t_{0.95}=\frac{|a|}{\frac{\sqrt{\frac{1}{N-2}<(x-{\bar{x}})^2>}}{\sqrt{<(t-{\bar{t}})^2>}}}>1.65. \end{aligned}$$

#### Computation of the joint histograms

We use joint histograms to assess the functional relation between two variables *y* and *x*. In our work we assess: (i) salinity trends (*y*) as function of the averaged salinity (*x*). This is shown in the bottom panel of Fig. 2, and; (ii) differences between surface and near surface salinity trends (*y*) as a function of trends of mixed layer depth, sea surface temperature and wind speed (*x*). This is shown in the top, mid and bottom panels respectively of Fig. 4. All the trends used in these histograms are statistically significant, with a significance larger than 0.95. Then, for each one of the joint histograms, we represent the average of the variable *y* at each bin of *x*: $\mu _{y|x_i}$. Besides, we represent the $95\%$ confidence interval associated with the mean given by:

4 $$\begin{aligned} \left( \mu _{y|x_i}-1.96\frac{\sigma _{y|x_i}}{\sqrt{N_i}}, \mu _{y|x_i}+1.96\frac{\sigma _{y|x_i}}{\sqrt{N_i}}\right) , \end{aligned}$$

where $\sigma _{y|x_i}$ is the standard deviation of the variable *y* at each bin of $x_i$, and $N_i$ the number of *y* values that have been averaged in the bin $x_i$.

The joint histograms are computed in each of the analyzed cases as follows:

- Bottom panel in Fig. 2: Mean salinity bins of 0.5 psu between 32 and 38 psu; Salinity trends between $-0.04$ and 0.04 (psu/y).
- Top panel in Fig. 4: MLD trend bins of 0.25 (m/y) between $-2$ and 2 (m/y); differences between SSS and NSS trends, from $-0.1$ to 0.1 (psu/y).
- Mid panel in Fig. 4: SST trend bins of 0.05 ($^\circ$/y) between $-0.5$ and 0.5 ($^\circ$/y); differences between SSS and NSS trend, from $-0.1$ to 0.1 (psu/y).
- Bottom panel in Fig. 4: WS trend bins of 0.05 (m/s/y) between $-0.25$ and 0.25 (m/s/y); differences between SSS and NSS trends in, from $-0.1$ to 0.1 (psu/y).

Note that the histograms in Fig. 4 are computed with data from the region $(40^\circ$ S, $40^\circ$ N).

## Supplementary Information

Supplementary Information.
