# Supplementary material for: Increasing stratification as observed by satellite sea surface salinity measurements
Source: Sci Rep. 2022 Apr 15;12:6279. doi: 10.1038/s41598-022-10265-1 (PMC9012883; doi:10.1038/s41598-022-10265-1)
Supplement: Supplementary file 1 — Supplementary Information. [file 41598_2022_10265_MOESM1_ESM.pdf]

Increasing stratification as observed by Satellite Sea Surface Salinity measurements.

October 2021

E. Olmedo[(1)(*)], A. Turiel[(1)], V. González-Gambau[(1)], C. González-Haro[(1)], A. García-Espriu[(1)], C. Gabarró[(1)], M. Portabella[(1)], I. Corbella[(2)], M. Martin-Neira[(3)], M. Arias[(1)], R. Catany[(4)], R. Sabia[(5)], R. Oliva[(6)], K. Scipal[(3)]

[(*)] Corresponding author: Estrella Olmedo, olmedo@icm.csic.es

[(1)] Barcelona Expert Center (BEC), Institute of Marine Sciences (ICM) and Consejo Superior de Investigaciones Científicas (CSIC), P. Marítim de la Barceloneta, 37-49, 08003 Barcelona, Spain

[(2)] Barcelona Expert Center (BEC), Universitat Politècnica de Catalunya (UPC), Barcelona, Spain

[(3)] European Space Agency, ESTEC, Noordwijk,The Netherlands

[(4)] Argans UK Ltd., Derriford, PL6 8BX Plymouth, U.K

[(5)] Telespazio-UK , for ESA-ESRIN, Largo Galileo Galilei 1, 00044 Frascati, Italy

[(6)] Zenithal Blue Technologies, 08023 Barcelona, Spain

1 SUPPLEMENTARY MATERIAL

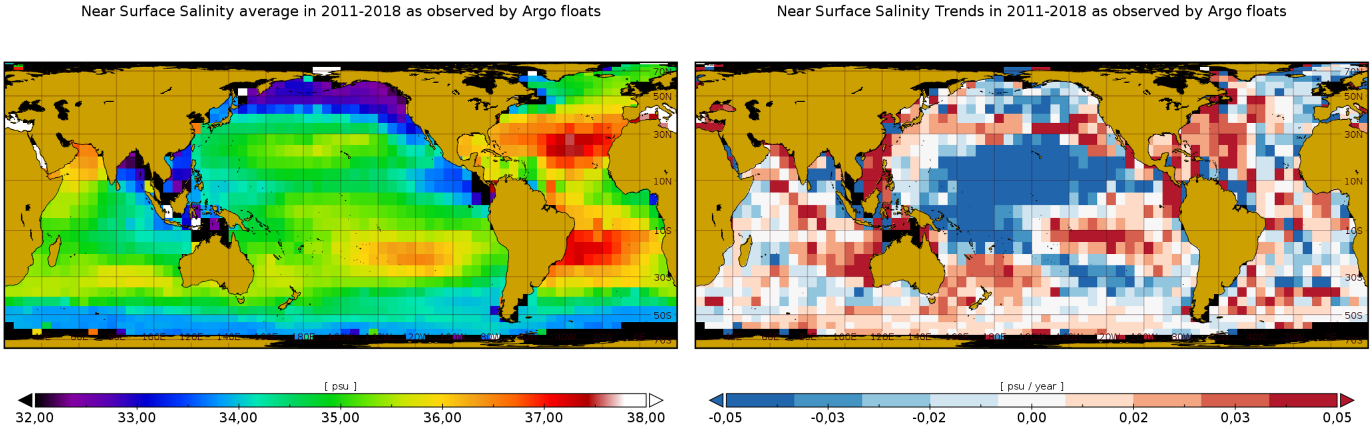

Figure 1: Left: Near-surface salinity average (left) and linear trend (right) in 2011-2018 as observed by Argo floats. Maps are plotted with Panoply v 4.12.0 (https://www.giss.nasa.gov/tools/panoply/).
